# Enhanced lysosome biogenesis ameliorates neurodegenerative diseases

**DOI:** 10.18632/aging.204389

**Published:** 2022-11-14

**Authors:** Wenlong Xue, Yang Li

**Affiliations:** 1 Department of Pharmacology, State Key Laboratory of Medical Neurobiology and MOE Frontiers Center for Brain Science, Key Laboratory of Metabolism and Molecular Medicine, Ministry of Education, School of Basic Medical Science, Fudan University, Shanghai, China

**Keywords:** lysosome-enhancing compounds, lysosome biogenesis, TFEB, neurodegenerative diseases

Autolysosomes are formed by the fusion of autophagosomes and lysosomes, they maintain the cellular homeostasis as the scavengers to digest the cytosolic cargos, including lipids, proteins, glycogen, and even damaged organelles. Autophagosomes are double-membrane structure organelles. They generate from membrane nucleation, then expand and bend into a spherical shape by taking up other organelles’ membranes, such as plasma membrane, mitochondria, and endoplasmic reticulum. In this process, biological macromolecules and damaged organelles are sequestered into the autophagosomes. Lysosomes are single-membrane and acidified organelles. The mature lysosomes have the acidic lumen (pH 4.5-5.0) to provide a microenvironment for lysosomal hydrolase activity. To achieve the ultimate stage of cargo degradation, the lysosomes fuse the outer membrane of autophagosomes to form autolysosomes, and then disintegrated the inner membrane of autophagosomes and cargos. Autolysosomal dysfunctions lead to significantly declined degradation efficiency and result in the accumulation of macromolecules or damaged organelles, which subsequently cause metabolism dysregulation and aggravate the pathogenesis of human diseases.

Neurodegenerative diseases are characterized by impaired cognitive function, and aging is one of the risk factors of pathogenesis of neurodegeneration. Neurodegenerative diseases include Alzheimer’s disease (AD), Parkinson’s disease (PD), Huntington’s disease (HD), Amyotrophic Lateral Sclerosis (ALS), and Lewy Body Dementia (LBD). According to recent studies, autolysosomal dysfunction aggravates the pathogenesis of these neurodegenerative diseases. In Tg2576/TRGL mice, a mouse model of AD, the deficiency of autolysosomal acidification occurs before Aβ accumulation [1]. These results demonstrate that autolysosomal dysfunction further aggravates protein accumulation, but not completely caused by deposits of toxic protein aggregates. In PD, autolysosome functions are also impaired, which further result in α-synuclein accumulation and accelerate PD progression. Interestingly, PD-risk protein TMEM175, a “K^+^ leak” channel in lysosomes, also plays a critical role in mediating the lysosomal H^+^ leak. Its deficiency causes lysosomal hyperacidification and results in α-synuclein aggregation [2]. In addition, Genetic mutations also promote the pathogenesis of neurodegenerative diseases. Microtubule-associated protein tau (MAPT) encodes the protein tau, its mutation causes autophagy-lysosomal pathway dysfunction and impaired lysosomal degradation, leading to disrupted degradation of protein aggregates [3]. TMEM106B, a lysosomal transmembrane protein, variants in frontotemporal dementia shows TMEM106B expression increasing, and its overexpression causes lysosomal acidification and trafficking disorder. Besides, Calcium (Ca^2+^) homeostasis also should be attention. Autophagosomes fuse with lysosomes in a Ca^2+^-dependent manner to form autolysosomes. Ca^2+^ dysregulation blocks the formation of autolysosomes, which decreases the degradation efficiency of protein aggregate in AD patients [4]. Chromosome 9 open reading frame 72 gene (C9orf72) has been identified to associate with ALS, and its deficiency in mouse and human cells leads to swelling of the lysosomes and abnormal lysosomal acidification, which causes impaired autolysosomal degradation. C9orf72 mutation, resulting in decreased transcription and protein levels, may accelerate the pathogenesis of ALS [5]. In conclusion, enhancing the function of autolysosomes is a critical strategy to improve neurodegenerative diseases.

On the other hand, enhanced functions of autophagosomes and lysosomes could ameliorate neurodegenerative diseases. Thus, it is necessary to dissect the underlying regulatory mechanisms of autophagososme-lysosome systems. Considering autolysosomes are fused from autophagosomes and lysosomes, one of the strategies is enhancement of cargo recognition by autophagosomes. Several new strategies of protein degradation dependent on autolysosomes have been developed, for instance, AuTophagosome-TEthering Compound (ATTEC), AUtophagy-TArgeting Chimera (AUTAC), LYsosome-TArgeting Chimera (LYTAC), and AUTOphagy-TArgeting Chimera (AUTOTAC) technologies [6].

The second strategy is enhancement of cargo degradation via lysosomes, so it is critical to promote lysosome biogenesis. Lysosome biogenesis is regulated by several key regulators on the transcriptional level, including transcription factor EB (TFEB), transcription factor E3 (TFE3), and repressor zinc finger protein with KRAB and SCAN domains 3 (ZKSCAN3). TFEB activation or ZKSCAN3 inactivation increases the expression of lysosomal genes and subsequently enhances lysosome biogenesis. We performed the compound screen based-on TFEB-EGFP expressing cell system and discover a group of LYsosome-Enhancing Compounds (LYECs) that can activate TFEB and promote lysosome biogenesis. HEP14, 5β-O-angelate-20-deoxyingenol, a compound isolated from *Euphorbia peplus* Linn, activates PKC thus inactivating GSK3β, which results in TFEB activation and promoting lysosome biogenesis. Notably, HEP14 also activates PKC-JNK/p38 MAPK to inactivate ZKSCAN3, which in turn enhances lysosome biogenesis [7]. In our recent study, LH2-051 is another member of LYECs. It directly binds with and inhibits dopamine transporters (DAT), and then promotes DAT translocation from plasma membranes onto lysosomal membranes. On lysosomal membranes, DAT translocation inhibits CDK9 activity, and promotes the release of CDK9 and TFEB from lysosomal membranes. This DAT-CDK9-TFEB axis promotes TFEB activation and lysosome biogenesis after LH2-051 treatment. Furthermore, injection of LH2-051 significantly improved behaviors of APP/PS1 mice in Y maze and Morris water maze tests, which was dependent on DAT-CDK9-TFEB signaling and LH2-051-induced lysosome biogenesis [8]. By the combination of the small-molecular compound library with our screen system, we also find that TSA, the well-known HDAC inhibitor, also promotes TFEB activation and lysosome biogenesis by increasing TFEB acetylation.

**Figure 1:**
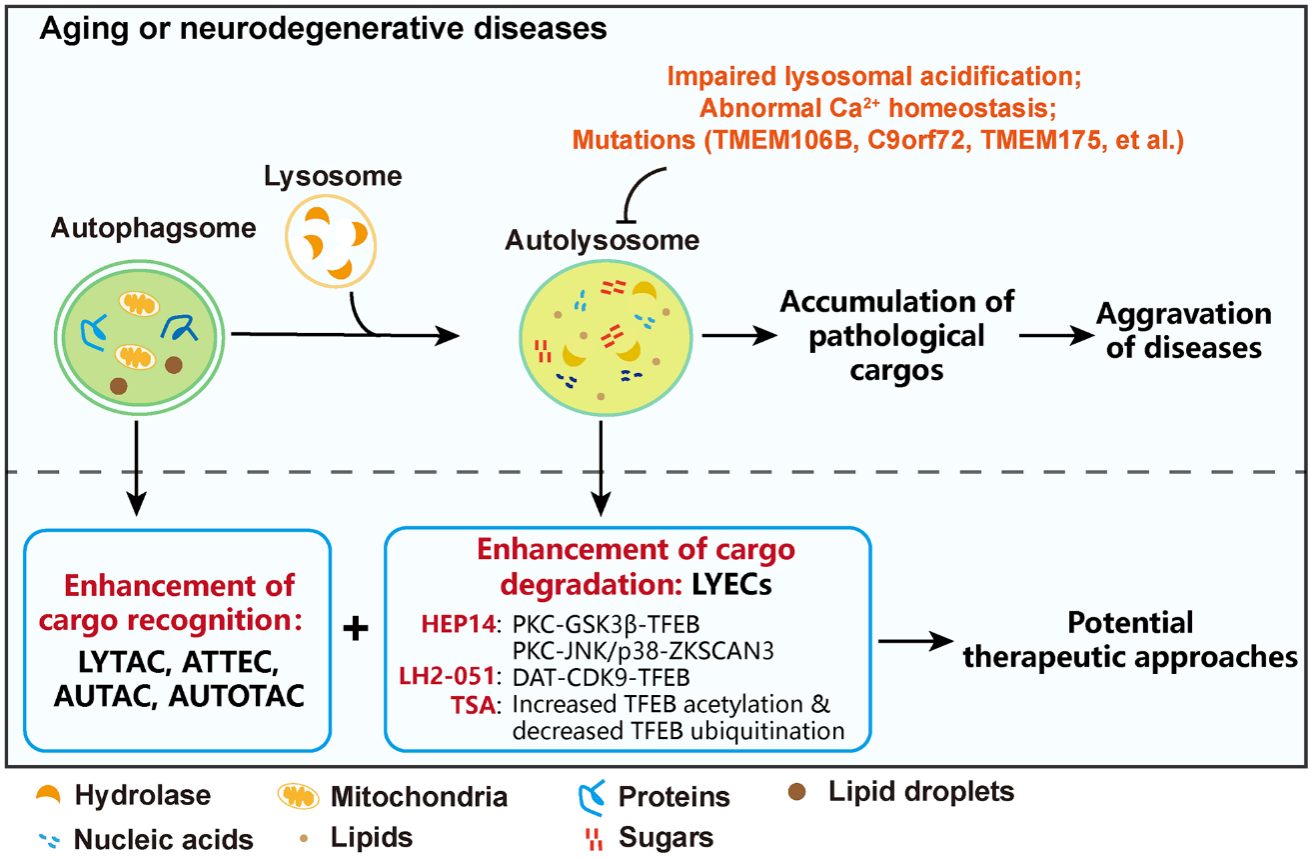
Combination of LYECs with ATTEC, AUTAC, LYTAC, or AUTOTAC is a potential therapeutic approach to ameliorate neurodegenerative diseases.

In future, the combination of LYECs with ATTEC, AUTAC, LYTAC, or AUTOTAC may have a synergetic effect and further enhance the cargo recognition and degradation by the autolysosome system. The drug cocktail therapy targeting protein aggregates may be a potential therapeutic approach to ameliorate neurodegenerative diseases (Figure 1).
